# Supplementary material for: Creation of flexible spin-caloritronic material with giant transverse thermoelectric conversion by nanostructure engineering
Source: Nat Commun. 2024 Mar 27;15:2184. doi: 10.1038/s41467-024-46475-6 (PMC10973454; doi:10.1038/s41467-024-46475-6)
Supplement: Supplementary file 1 — Supplementary Information [file 41467_2024_46475_MOESM1_ESM.pdf]

Supplementary Information

Creation of flexible spin-caloritronic material with giant transverse thermoelectric conversion by nanostructure engineering

Ravi Gautam[1], Takamasa Hirai[1], Abdulkareem Alasli[2], Hosei Nagano[2], Tadakatsu Ohkubo[1], Ken-ichi Uchida[1*], Hossein Sepehri-Amin[1*]

[1]*National Institute for Materials Science, Tsukuba, 305-0047, Japan*
[2]*Department of Mechanical Systems Engineering, Nagoya University, Nagoya 464-8601, Japan*
*Corresponding authors. *Email address:* h.sepehriamin@nims.go.jp (H. Sepehri-Amin) and uchida.kenichi@nims.go.jp (K. Uchida).

Supplementary Note

Thermal Diffusivity

Supplementary Fig. 5 illustrates a schematic of the setup consisting of an infrared camera, a diode laser, a function generator, and a LIT system. Supplementary Fig. 6 shows the ϕ images and the corresponding fitted data along the longitudinal axis of the samples (θ = 0° - 180°). The ϕ images indicate that clear laser-induced thermal modulation signals appear on the surface of the samples. The ϕ distribution has the expected relatively uniform circular shape due to the heat diffusion from the heat point. Furthermore, the fitted experimental data show the linear dependency of ϕ with r and good agreement with equation (1) (see Methods).

Specific Heat

Supplementary Fig. 8 shows the specific heat C_p at room temperature of the Nanomet sample with varying annealing temperatures measured by the differential scanning calorimetry (DSC). Typically, the amorphous phase has a higher heat capacity than the crystalline phase due to the presence of more vibrational modes in the amorphous material. This leads to a greater energy storage capacity and, therefore, a higher specific heat, which is consistent with the trend seen in the specific heat with annealing temperature.

Anomalous Nernst/Ettingshausen Effect

Supplementary Fig. 10 shows the lock-in thermography measurement of AEE for the annealed Nanomet samples, where an apparent current-induced temperature modulation uniformly appeared across the entire surface of the ribbons. Although some samples displayed slight variations in A_{odd} values between the left-side and right-side wires due to the difference in wire width, the lock-in amplitude per unit current density, A_{odd}/j_c, is the same. The ϕ_{odd} value of the left-side (right-side) wire was observed to be ~0° (~180°) with a charge current applied to the $+x$-direction ($-x$ direction) and a magnetic field in the $+y$-direction, satisfying the behavior of the AEE-induced temperature modulation for samples showing positive S_{ANE} and Π_{AEE}[1-3]. Here, the definition of the x and y direction is shown in Supplementary Fig. 10. The f dependence of A_{odd}/j_c showed almost no change, indicating the AEE-induced temperature modulation reaches the steady state in the present f range.

Nanostructure engineering of Fe-based amorphous materials with high Cu content

To validate the efficacy of our nanostructure engineering approach in tailoring ANE, we prepared another Fe-based amorphous material with a higher Cu content using the melt-spinning technique. The composition of this material was estimated using inductively coupled plasma optical emission spectrometry (ICP-OES) and was found to be $Fe_{80.5}Si_{3.8}B_{13.6}Cu_{1.5}C_{0.6}$ (at.%). DSC analysis of the as-quenched sample reveals the crystallization temperature of the α-Fe(Si) phase at 656 K, as depicted in Supplementary Fig. 12a. The as-quenched ribbons were annealed at various temperatures (626 K, 646 K, and 656 K) to facilitate the formation of high-density nano-sized Cu-clusters. X-ray diffraction (XRD) analysis, presented in Supplementary Fig. 12b, indicates the presence of an amorphous phase for the samples annealed below 646 K, while the sample annealed at 656 K exhibited the formation of α-Fe(Si) crystalline phase. Atom probe tomography (APT) analysis of both as-quenched and 646 K annealed samples was carried out to study the formation of Cu-clusters, as illustrated in Supplementary Fig. 13. Due to the higher Cu content, APT elemental mapping of the as-quenched sample reveals the presence of Cu-clusters in an amorphous matrix. Annealing at 646 K led to a fourfold increase in the number density of Cu-clusters. Notably, the increase in Cu-cluster density also resulted in the enhancement of the S_{ANE} value from 2.63 μVK^{-1} to 3.48 μVK^{-1}, as demonstrated in Supplementary Fig. 14. These findings provide additional evidence that nanostructure engineering in ribbons with different compositions can indeed result in an increase in ANE.

An increase in Cu-content from $Fe_{84.7}Si_{2.8}P_{3.8}B_{7.8}Cu_{0.7}C_{0.2}$ to $Fe_{80.5}Si_{3.8}B_{13.6}Cu_{1.5}C_{0.6}$ led to an increase in Cu cluster density from 7.3×10^{23} m^{-3} to 9.05×10^{23} m^{-3}, respectively. Importantly, the high Cu content Fe-based amorphous material holds merits in terms of the power factor and figure of merit values due to enhanced electrical conductivity, as shown in Supplementary Fig. 15. However, the high Cu content sample shows a smaller S_{ANE} value, which can be attributed to alterations in the composition of the matrix amorphous phase. Therefore, ANE can be further amplified by employing compositional as well as nanostructure engineering.

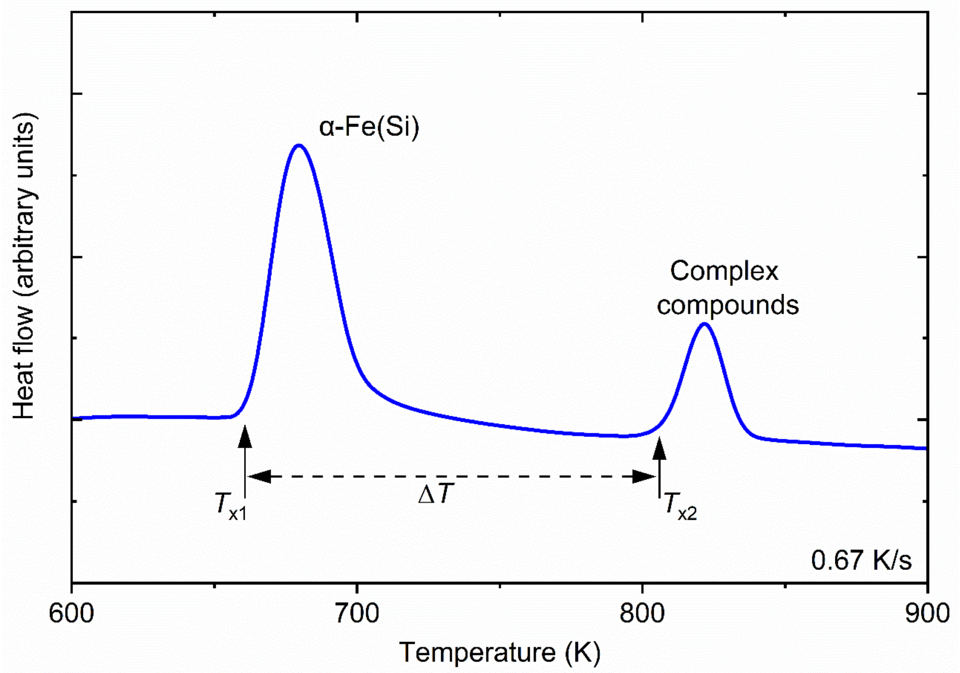

Supplementary Fig. 1 | DSC curve of the as-quenched melt-spun Nanomet ribbon.

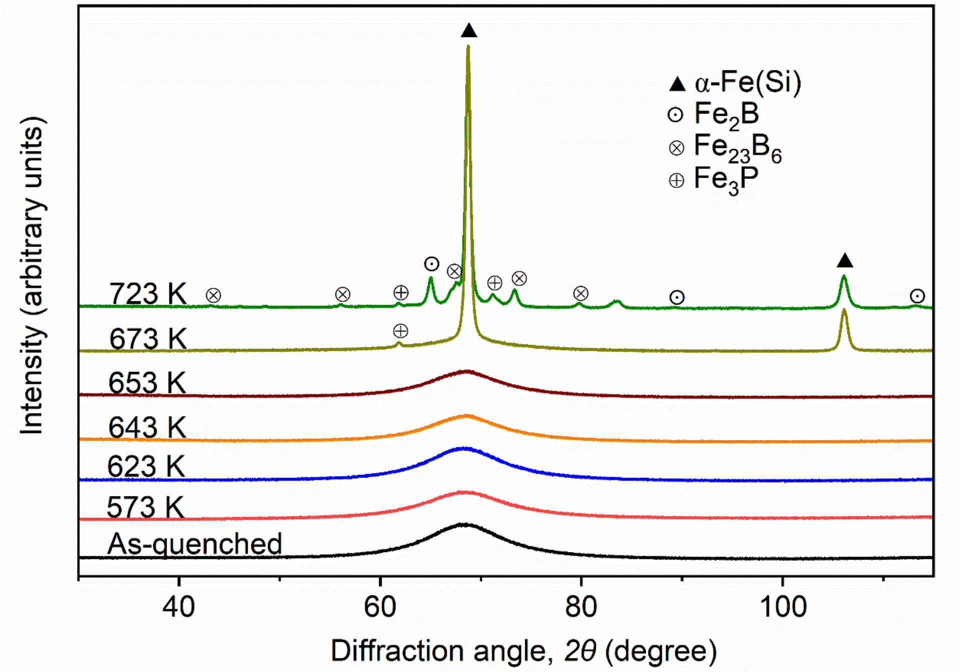

Supplementary Fig. 2 | XRD patterns of the annealed Nanomet samples.

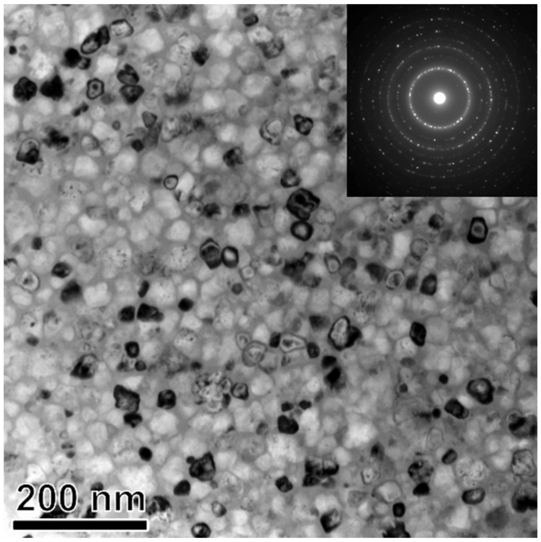

Supplementary Fig. 3 | Bright field TEM image of the sample annealed at 673 K with an inset of the selected area electron diffraction pattern.

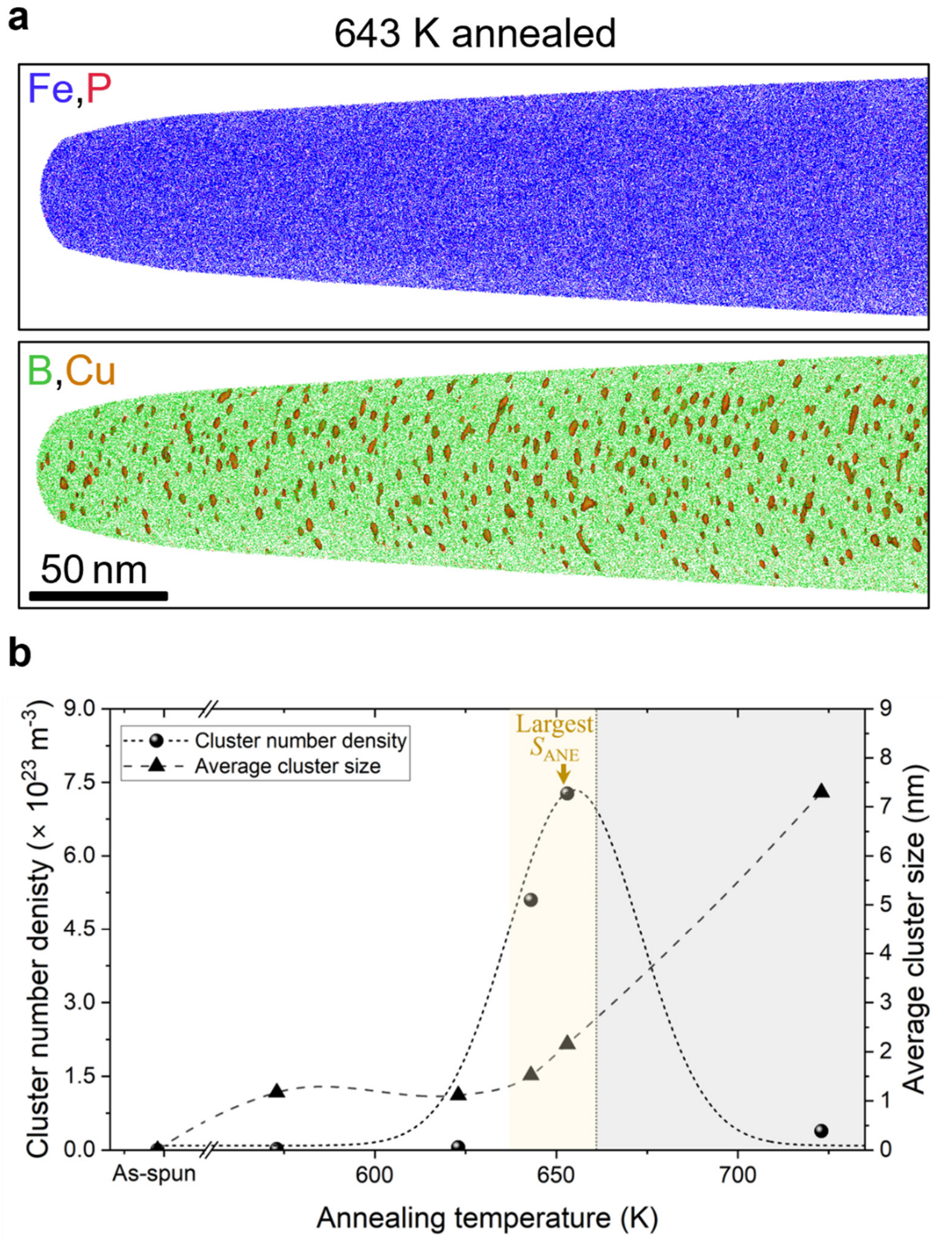

Supplementary Fig. 4 | APT analysis of Nanomet sample annealed at 643 K and variation in the number density and size of Cu clusters for Nanomet samples as a function of annealing temperature. a, APT elemental maps of Fe (blue), P (red), B (green), and Cu (orange) for Nanomet sample annealed at 643 K. **b,** Annealing temperature dependence of the Cu-cluster number density and average Cu-cluster size. The white region indicates the presence of Cu-clusters in an amorphous matrix, with the grey dotted line denoting the onset of primary crystallization and grey regions signify the presence of α-Fe crystalline phase and Cu-clusters in an amorphous matrix. The light gold region represents the annealing temperature associated with a high-density region of Cu-clusters.

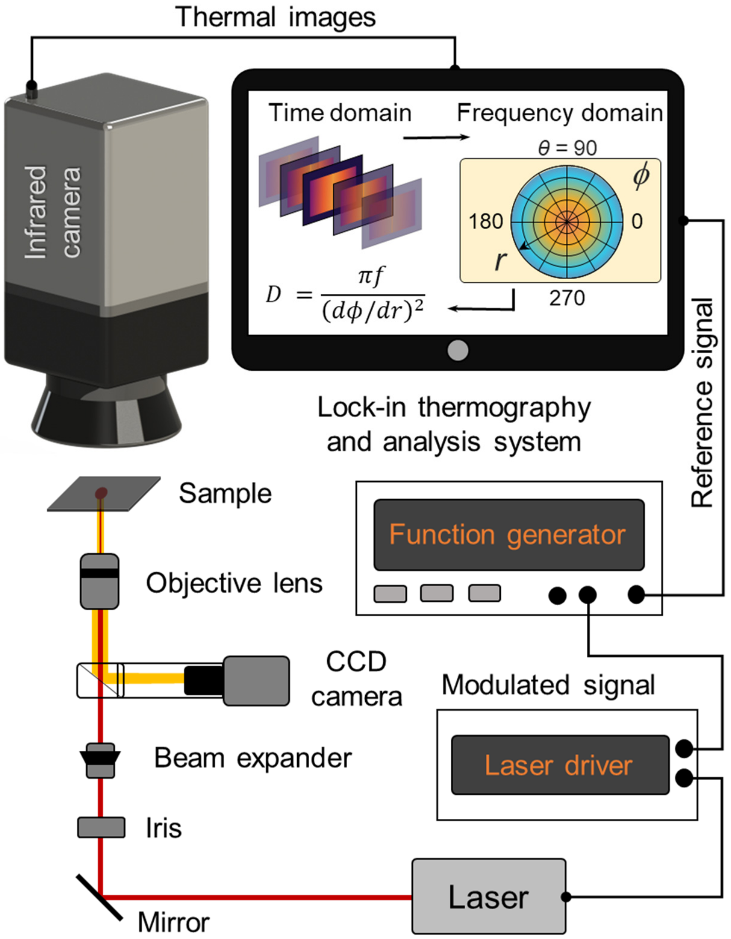

Supplementary Fig. 5 | A Schematic of lock-in-thermography (LIT)-based thermal diffusivity measurement technique. The sample is located in the focal plane of the infrared camera. The diode laser emits a modulated beam at frequency f, which is focused on the backside of the sample by the optical setup. The thermal diffusivity D is estimated from the phase ϕ correlation to distance r at specific f. The angular distribution of D is obtained by revolving the analysis around the laser-heat point at angles θ.

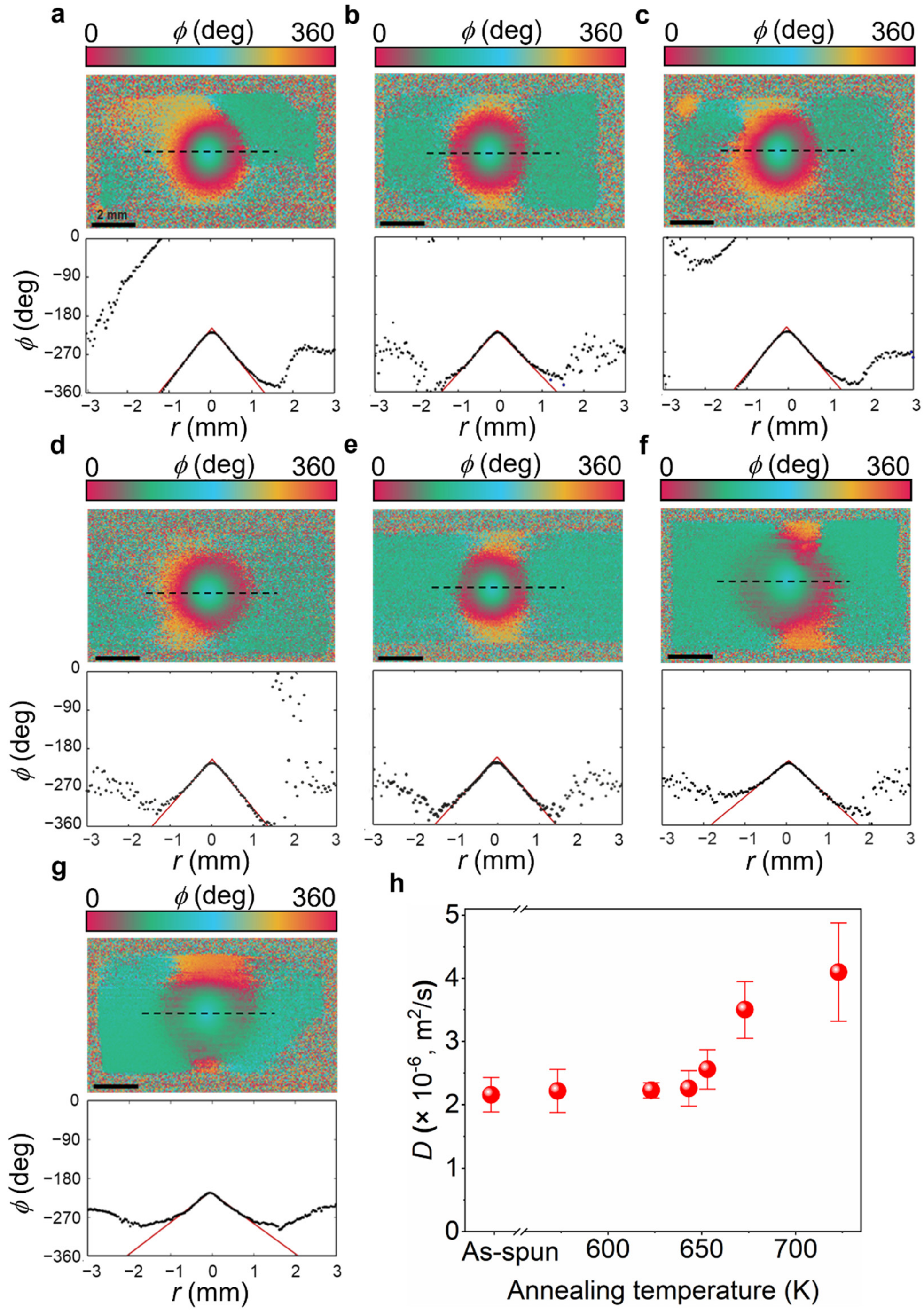

Supplementary Fig. 6 | Thermal diffusivity measured of the annealed Nanomet samples. ϕ images with corresponding line profiles along the longitudinal direction (dashed line) of respectively **a,** as-quenched, **b,** annealed at 573 K, **c,** 623 K, **d,** 643 K, **e,** 653 K, **f,** 673 K and **g,** 723 K at f = 3 Hz and laser power P = 10 mW. r = 0 in the line profiles was determined by the position of the laser heating. The solid red lines in **a-g** represent the fitting results using $D = \pi f/(d\phi/dr)^2$. **h,** D values of the annealed Nanomet samples.

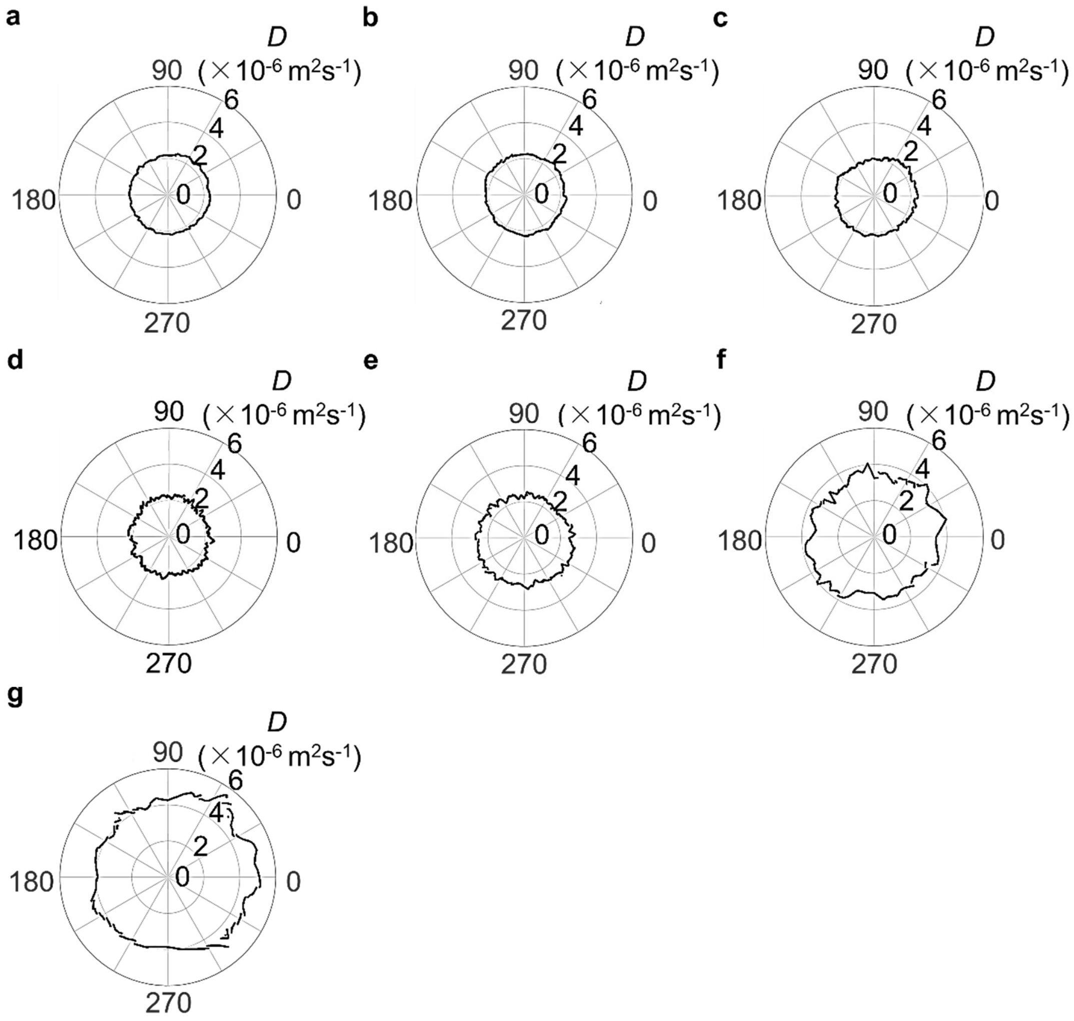

Supplementary Fig. 7 | Investigation of angular distribution of thermal diffusivity for the annealed Nanomet samples. Angular distribution of D ($\times 10^{-6}$ m^{2}s^{-1}) measured at $f = 3$ Hz and $T = 300$ K for **a,** as-quenched sample, **b,** annealed at 573 K, **c,** 623 K, **d,** 643 K, **e,** 653 K, **f,** 673 K and **g,** 723 K. The centre of the graph is located on the position of the laser heating while 0° - 180° and 90° - 270° axis represent respectively the longitudinal and the transverse axes of the sample.

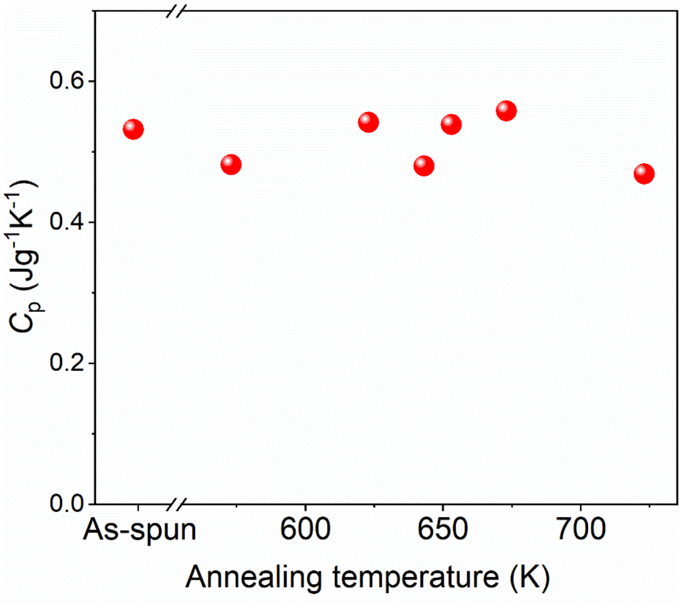

Supplementary Fig. 8 | Specific heat C_p as a function of annealing temperature for the Nanomet samples.

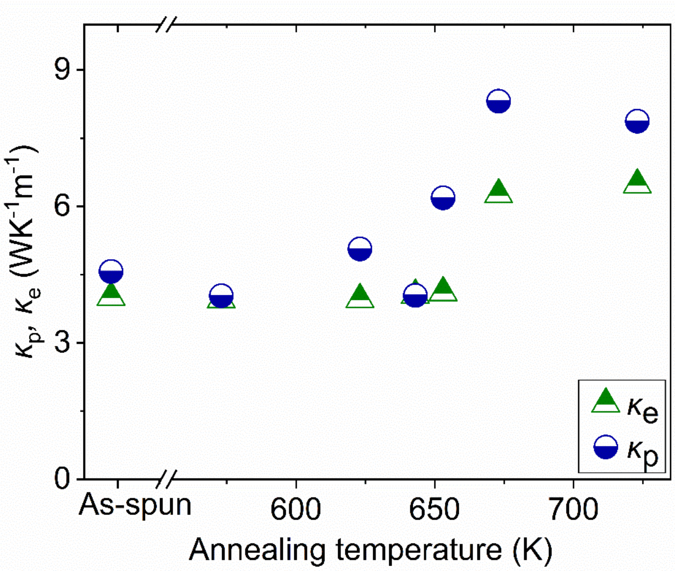

Supplementary Fig. 9 | Phonon thermal conductivity κ_p and electron thermal conductivity κ_e as a function of annealing temperature for the Nanomet samples.

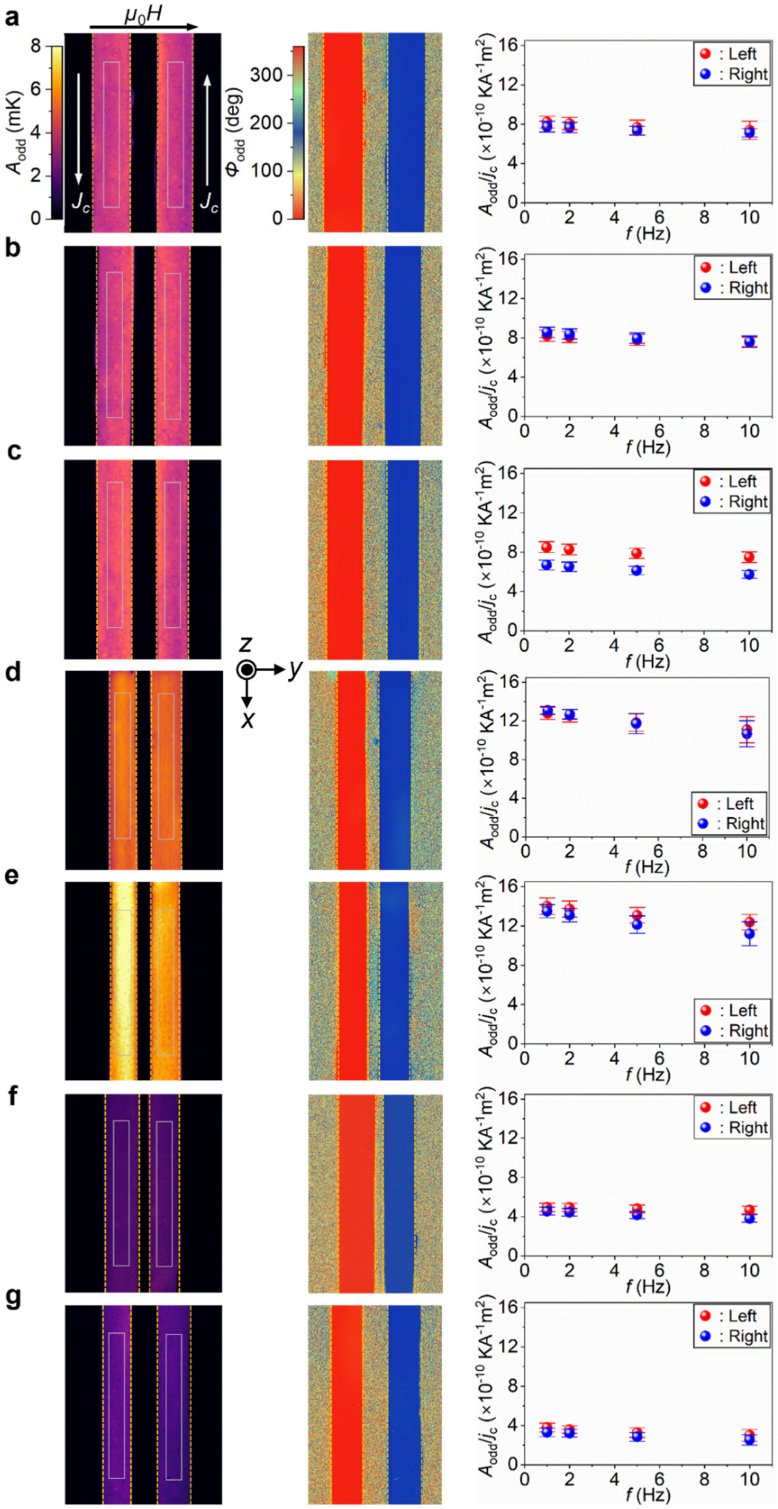

Supplementary Fig. 10 | Lock-in thermography measurement of AEE for the annealed Nanomet samples. Field-odd component of lock-in amplitude (A_{odd}) and phase (ϕ_{odd}) images at $\mu_0 H = 1.0$ T and the corresponding frequency f dependence of $A_{\mathrm{odd}}/j_{\mathrm{c}}$ for **a,** as-quenched, **b,** annealed at 573 K, **c,** 623 K, **d,** 643 K, **e,** 653 K, **f,** 673 K and **g,** 723 K samples. $\mathbf{J}_{\mathrm{c}}$ denotes the charge current applied to the sample with charge current density j_{c}. The values and error bars in the rightmost graphs represent the average and standard deviation of A_{odd} on the plot area shown by grey rectangular boxes in the A_{odd} images, respectively.

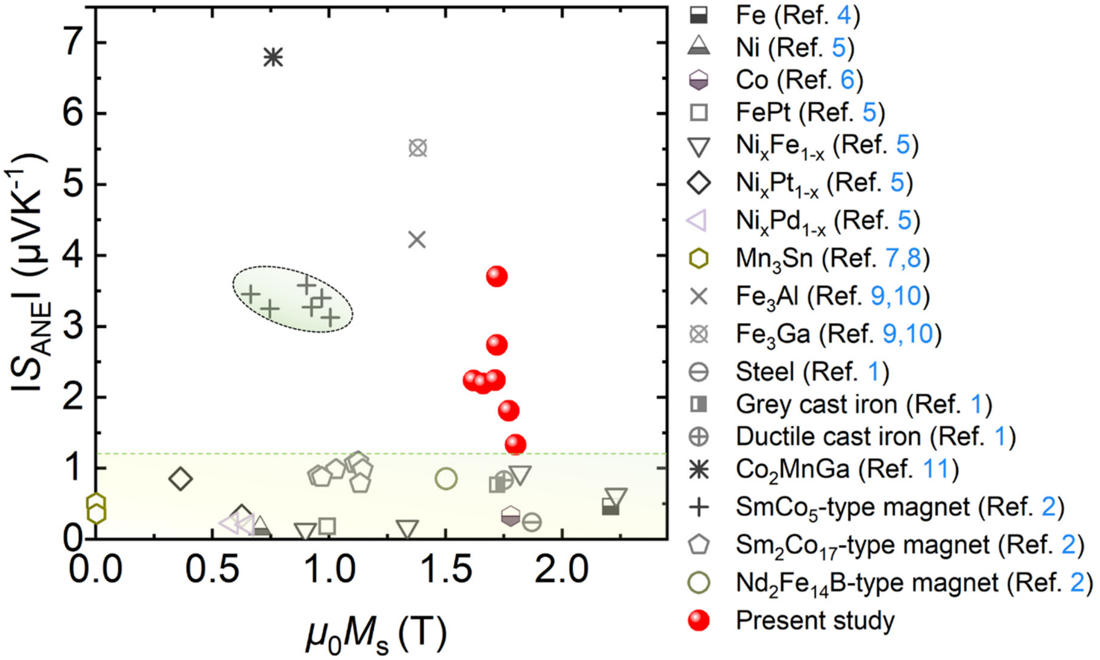

Supplementary Fig. 11 | Comparison of the anomalous Nernst effect for spin-caloritronic materials. Comparison of the absolute values of the anomalous Nernst coefficient ($|S_{ANE}|$) with the magnetization ($\mu_0 M_s$) for various bulk spin-caloritronic materials measured at room temperature[10]. The green dotted line represents the upper limit of the $|S_{ANE}|$ signals in conventional materials.

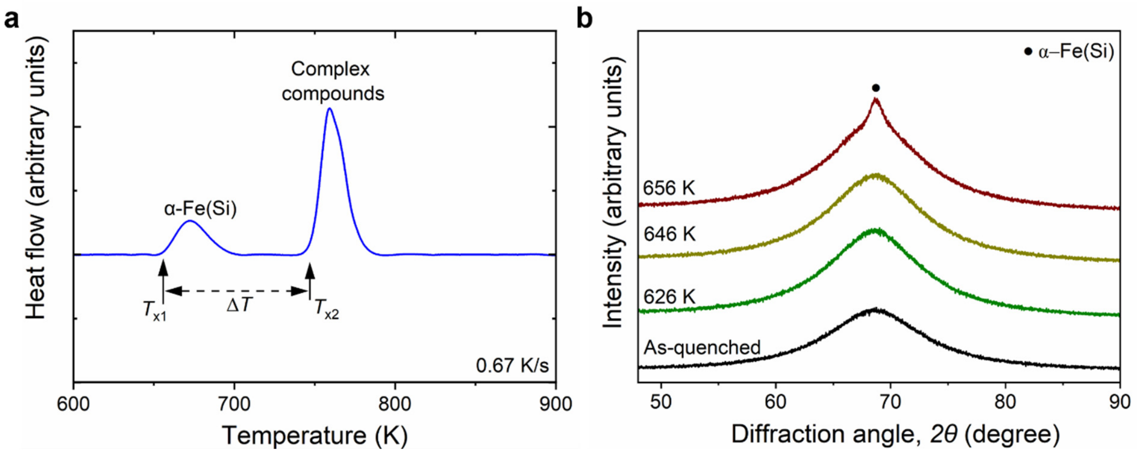

Supplementary Fig. 12 | DSC and XRD analysis of high Cu content Fe-based amorphous material. a, DSC curve of the as-quenched melt-spun ribbon and **b,** XRD pattern of high Cu content Fe-based amorphous samples ($Fe_{80.5}Si_{3.8}B_{13.6}Cu_{1.5}C_{0.6}$ at.%) annealed at 646 K and different time.

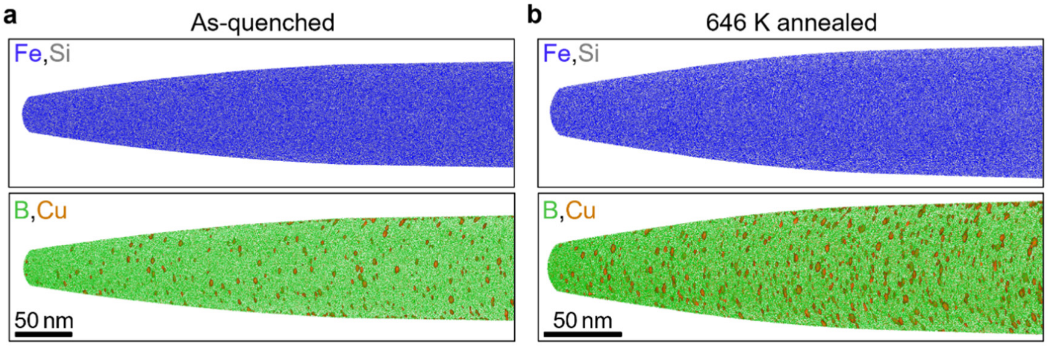

c

	As-quenched	646 K annealed
Average cluster size (nm)	2.02	2.45
Cluster number density ($\times$ 10^{23} m^{-3})	1.99	9.05

Supplementary Fig. 13 | APT analysis of high Cu content Fe-based amorphous material. a,b, APT elemental maps depict the distribution of Fe (blue), Si (grey), B (green), and Cu (orange) in the as-quenched sample (**a**) and sample annealed at 646 K (**b**). **c,** Estimated cluster size and number density of both as-quenched and annealed samples.

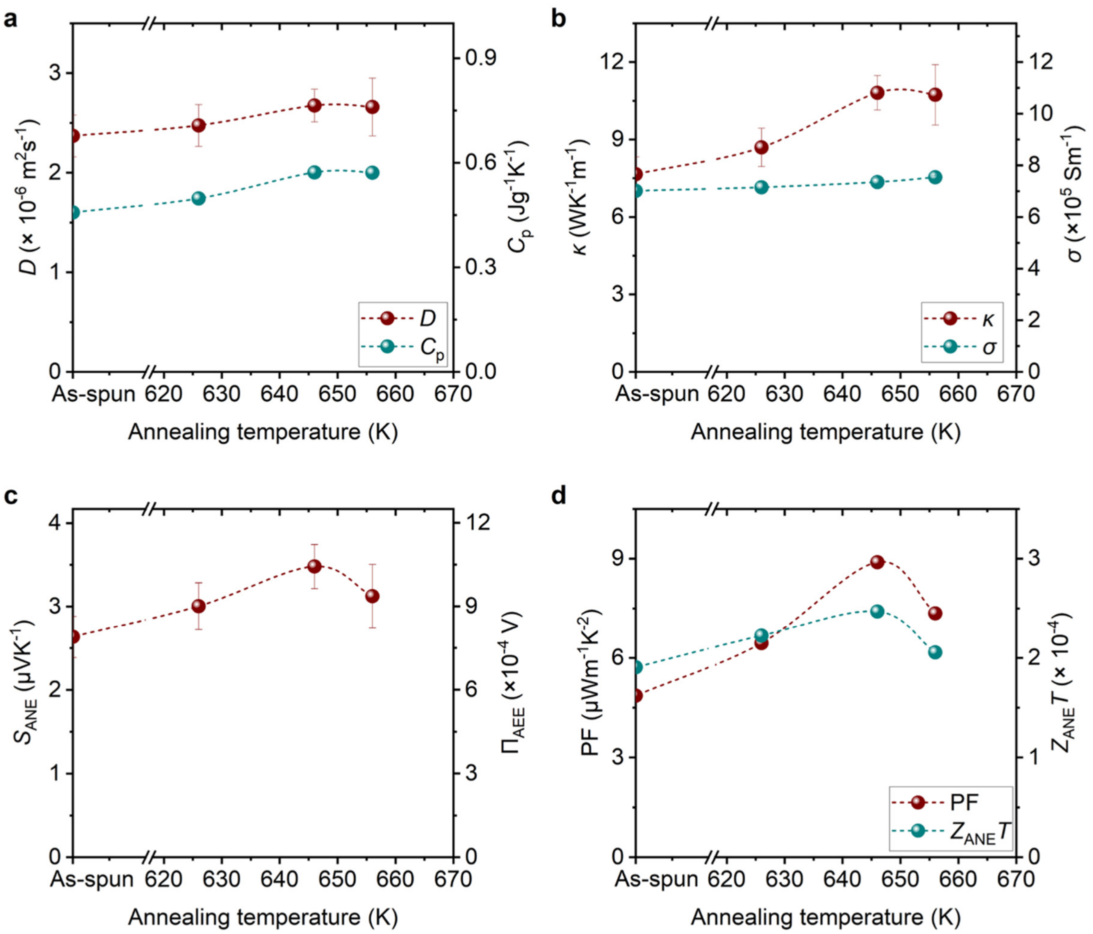

Supplementary Fig. 14 | Transport properties of high Cu content Fe-based amorphous samples. a-d, Annealing temperature dependence of the thermal diffusivity D and specific heat C_p (**a**), thermal conductivity κ and electrical conductivity σ (**b**), anomalous Nernst coefficient S_{ANE} and the corresponding anomalous Ettingshausen coefficient Π_{AEE} estimated using the Onsager reciprocal relation at 300 K (**c**), and power factor (PF) and dimensionless figure of merit for ANE $Z_{ANE}T$ at 300 K (**d**) for the high Cu content Fe-based amorphous samples.

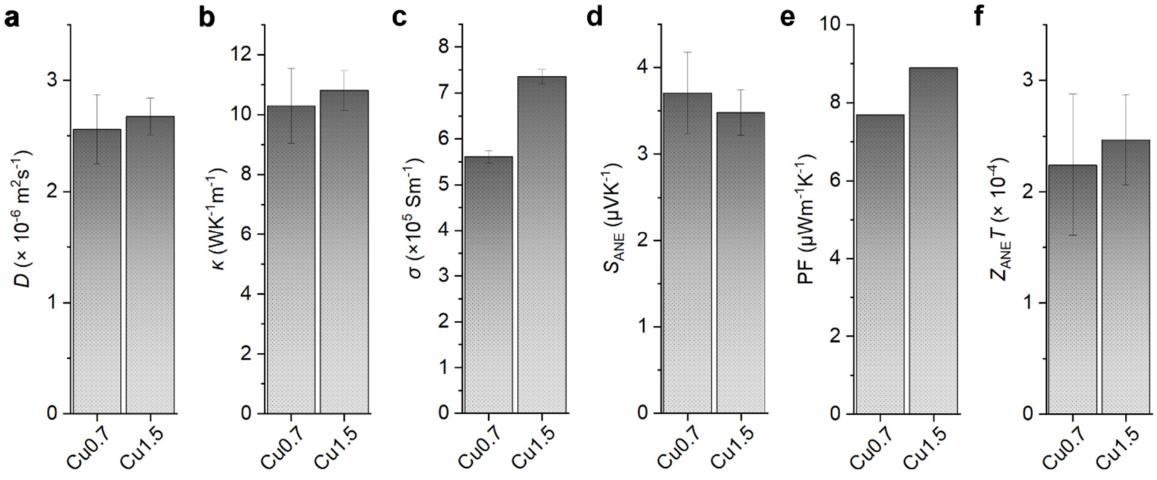

Supplementary Fig. 15 | Comparison of properties between low and high Cu content Fe-based amorphous samples. a-f, Thermal diffusivity D (**a**), thermal conductivity κ (**b**), electrical conductivity σ (**c**), anomalous Nernst coefficient S_{ANE} (**d**), Power factor (PF) (**e**), and dimensionless figure of merit for ANE $Z_{\mathrm{ANE}}T$ at 300 K (**f**) between low and high Cu content Fe-based amorphous samples. Samples with the highest ANE values are chosen for this comparison.

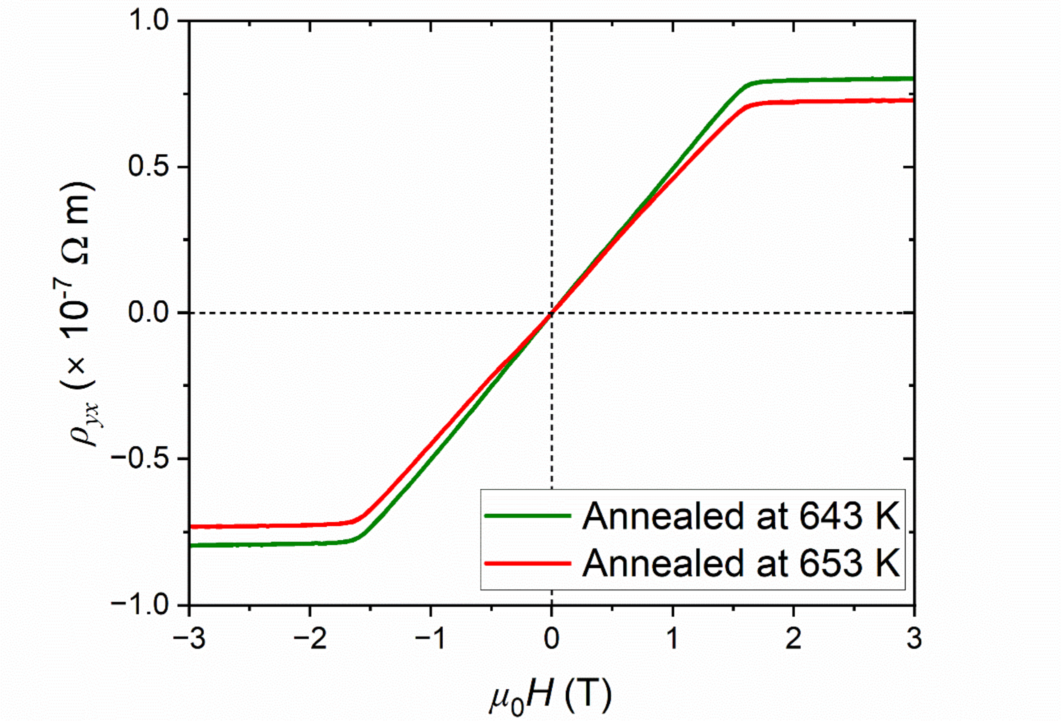

Supplementary Fig. 16 | Magnetic field dependence of the transverse electrical resistivity ρ_{yx} measured at room temperature for the Nanomet samples annealed at 643 K and 653 K.

References

1. Nagasawa, R. *et al.* Anomalous Ettingshausen effect in iron-carbon alloys. *Appl. Phys. Lett.* **121**, 062401 (2022).

2. Miura, A. *et al.* Observation of anomalous Ettingshausen effect and large transverse thermoelectric conductivity in permanent magnets. *Appl. Phys. Lett.* **115**, 222403 (2019).

3. Miura, A. *et al.* High-temperature dependence of anomalous Ettingshausen effect in $SmCo_5$-type permanent magnets. *Appl. Phys. Lett.* **117**, 082408 (2020).

4. Butler, E. H. & Pugh, E. M. Galvano- and thermomagnetic phenomena in iron and nickel. *Phys. Rev.* **57**, 916–921 (1940).

5. Miura, A., Iguchi, R., Seki, T., Takanashi, K. & Uchida, K. Spin-mediated charge-to-heat current conversion phenomena in ferromagnetic binary alloys. *Phys. Rev. Mater.* **4**, 1034409 (2020).

6. Smith, A. W. The transverse thermomagnetic effect in nickel and cobalt. *Phys. Rev. (Series I)* **33**, 295–306 (1911).

7. Ikhlas, M. *et al.* Large anomalous Nernst effect at room temperature in a chiral antiferromagnet. *Nat. Phys.* **13**, 1085–1090 (2017).

8. Li, X. *et al.* Anomalous Nernst and Righi-Leduc Effects in Mn_3Sn: Berry curvature and entropy flow. *Phys. Rev. Lett.* **119**, 056601 (2017).

9. Sakai, A. *et al.* Iron-based binary ferromagnets for transverse thermoelectric conversion. *Nature* **581**, 53–57 (2020).

10. Uchida, K., Zhou, W. & Sakuraba, Y. Transverse thermoelectric generation using magnetic materials. *Appl. Phys. Lett.* **118**, 140504 (2021).

11. Zhou, W., Miura, A., Hirai, T., Sakuraba, Y. & Uchida, K. Seebeck-driven transverse thermoelectric generation in magnetic hybrid bulk materials. *Appl. Phys. Lett.* **122**, 062402 (2023)
